# Supervised exercise training improves cardiorespiratory fitness and reduces perioperative risk in peripheral artery disease patients with intermittent claudication

**DOI:** 10.1308/rcsann.2022.0169

**Published:** 2023-05-02

**Authors:** D Lanéelle, M Hughes, BS Stacey, M Bashir, IM Williams, MH Lewis, DM Bailey

**Affiliations:** ^1^ Neurovascular Research Laboratory, Faculty of Life Sciences and Education, University of South Wales, Pontypridd, UK; ^2^ Vascular Medicine Unit, Centre Hospitalier Universitaire de Caen Normandie, 14000 Caen, France; ^3^ Department of Physiotherapy, Prince Charles Hospital, Merthyr Tydfil, UK; ^4^ Department of Surgery, University Hospital Wales, Cardiff, UK

**Keywords:** Peripheral artery disease, Intermittent claudication, Exercise training, Cardiorespiratory fitness, Perioperative risk

## Abstract

**Introduction:**

This study examined to what extent supervised aerobic and resistance exercise combined with continued unsupervised exercise training improves cardiorespiratory fitness and corresponding perioperative risk in peripheral artery disease (PAD) patients with intermittent claudication.

**Methods:**

A total of 106 patients (77% male) were enrolled into the study, alongside 155 healthy non-PAD control participants. Patients completed supervised exercise therapy (aerobic and resistance exercises of the upper and lower limbs) twice a week for 10 weeks. Thereafter, 52 patients completed 12 weeks of an unsupervised tailored home-based exercise. Pain-free walking distance (PWD), maximum walking distance (MWD), peak oxygen uptake ($\dot{V}O_{2\mathrm{PEAK}}$) and perioperative risk were assessed before and after both exercise interventions.

**Results:**

Patients were highly unconditioned relative to healthy controls ($\dot{V}O_{2\mathrm{PEAK}}$=11.9 vs 24.2ml/kg/min, *p*=<0.001) with 91% classified as high perioperative risk (peak oxygen uptake <15ml/kg/min). Supervised exercise increased PWD (+44±81m, *p*=<0.001), MWD (+44±71m, *p*=<0.001) and $\dot{V}O_{2\mathrm{PEAK}}$ (+1.01±1.63ml/kg/min, *p*=<0.001) and lowered perioperative risk (91% to 85%, *p*=<0.001). When compared with supervised exercise, the improvements in PWD were maintained following unsupervised exercise (+11±91m vs supervised exercise, *p*=0.572); however, MWD and $\dot{V}O_{2\mathrm{PEAK}}$ decreased (−15±48m, *p*=0.030 and −0.34±1.11ml/kg/min, *p*=0.030, respectively) and perioperative risk increased (+3%, *p*=<0.001) although still below baseline (*p*=<0.001).

**Conclusions:**

Supervised aerobic and resistance exercise training and, to a lesser extent, unsupervised tailored exercise improves walking capacity and cardiorespiratory fitness and reduces perioperative risk in PAD patients with intermittent claudication.

## Introduction

Lower extremity peripheral artery disease (PAD) is a common disease, affecting an estimated 235 million people globally and associated with considerable morbidity and mortality.^1^ Leg pain induced by exercise and relieved with rest typifies the clinical diagnosis of intermittent claudication (IC).^2^ Lifestyle changes combined with exercise training remain a cornerstone of IC management and international guidelines recommend exercise training among first-line therapies for patients with symptomatic PAD.^3^ The benefits of both aerobic and resistance exercise are well known and include improvements in maximum walking distance (MWD, also known as absolute claudication distance), pain-free walking distance (PWD, also known as initial claudication distance), cardiorespiratory fitness (CRF),^4^ quality of life and mortality.^5,6^

It has also been shown that an increase in CRF, assessed by cardiopulmonary exercise testing (CPET), is associated with improved postoperative survival in patients scheduled for elective abdominal aortic aneurysm repair,^7^ reduced intensive therapy unit requirement and reduced hospital length of stay.^8,9^ Accordingly, a discriminatory peak oxygen uptake ($\dot{V}O_{2\mathrm{PEAK}}$) threshold value of <15ml/kg/min has been identified to categorise those at increased risk of morbidity and mortality in patients undergoing major noncardiac surgery.^10^ As a likely consequence of physical inactivity, muscle weakness and accompanying comorbidities,^11^ a recent meta-analysis identified a mean $\dot{V}O_{2\mathrm{PEAK}}$ of 15.7±2.8ml/kg/min in IC patients,^12^ yet their corresponding perioperative risk reduction in response to an exercise intervention has not been explored.

To address this knowledge gap, the present study sought to firstly determine CRF and perioperative risk in patients with IC relative to age-matched healthy controls, and second, to what extent a 10-week supervised aerobic and resistance exercise therapy followed by a 12-week unsupervised exercise therapy potentially improves functional measures of CRF and corresponding implications for perioperative risk stratification.

## Methods

### Ethical approval

Ethical approval was granted by Cwm Taf University Health Board (CT/838/17) and American Medical International (Texas, USA). All procedures were carried out in accordance with the Declaration of Helsinki of the World Medical Association,^13^ with written informed consent obtained from all participants and patients.

### Non-PAD controls

For the purposes of comparing baseline CRF, we recruited 155 consecutive non-PAD participants, attending the same hospital at the same time, who were participating in a cardiovascular screening programme due to the presence of risk factors. They were selected retrospectively based on a normal 12-lead electrocardiographic response to a functional diagnostic Bruce protocol graded exercise test to volitional exhaustion. All participants were asymptomatic without claudication and defined as sedentary since they did not engage in any form of recreational activity outside of everyday living.^14^

### Patients

The study cohort was drawn from a consecutive sample of vascular patients with symptomatic atherosclerotic disease who had undergone lower limb vascular imaging via Duplex ultrasound following presentation to their local hospital vascular unit (Royal Glamorgan Hospital, Llantrisant, UK). All patients were bipeds and were allocated to a supervised (hospital-based) exercise training intervention that consisted of a combination of aerobic and resistance-based exercises. Thereafter, patients were allocated to complete an unsupervised (home and community-based) aerobic exercise training intervention.

### Clinical history

Patients underwent a thorough medical examination in which demographic information, body mass index (BMI), vascular risk factors, comorbid conditions, claudication history, ankle/brachial index (ABI) and list of current medications were recorded. Obesity was defined by a BMI≥30kg/m^2^, and overweight as a BMI≥25kg/m^2^. Hypertension was defined by having at least one of the following conditions: a systolic blood pressure ≥140mmHg, a diastolic blood pressure ≥90mmHg, or currently prescribed antihypertensive medications. Dyslipidaemia was defined by having at least one of the following conditions: a cholesterol value ≥5.2mmol/l, a triglyceride value ≥1.7mmol/l, a low-density lipoprotein level ≥3.4mmol/l, a high-density lipoprotein level <1.0mmol/l in men and <1.3mmol/l in women, or currently prescribed lipid-lowering medications. Diabetes was defined by having at least one of the following conditions: a fasting blood glucose ≥7.0mmol/l or currently prescribed oral medication or insulin. A history of lower extremity revascularisation, smoking, cerebral vascular accidents (stroke or transischemic attacks), and previous angina were determined by self-report, and a history of chronic obstructive pulmonary disease (COPD) was determined if the patient reported that they had been diagnosed by their physician. The presence of uni/bilateral disease was noted by the consultant radiologist including the number of segments affected. Only stenoses of ≥50% were considered radiologically significant.

### Inclusion criteria

Each patient was deemed suitable for exercise; diagnosed by an ABI ≤0.90 at rest, stenoses ≥50% by computed tomography angiography or Duplex ultrasound and relevant history. Each patient that chose to participate in the exercise therapy demonstrated an ability to exercise at a level appropriate for the group sessions, with IC being the main limiting factor to continue exercise. Patients were referred to the exercise clinic with additional input from the consultant cardiologist and senior physiotherapist following discussion with the consultant vascular surgeon. They were all suitable for surgery or endovascular intervention but at an early stage of the disease (stage II of Fontaine classification), and the consultant vascular surgeon stated that they could benefit from exercise therapy in the first instance.

### Exclusion criteria

Patients were excluded only for the following reasons: lower limb amputation or ulceration, stroke suffered within 6 months, acute coronary syndrome, coronary artery bypass surgery or endovascular angioplasty of coronary arteries within 12 months, aortic aneurysm, advanced kidney or liver failure, failure of the respiratory or circulatory system, active neoplastic disease, advanced chronic venous insufficiency, Buerger’s disease, uncontrolled hypertension, mental disease or dementia, failure to complete all of the baseline tests and failure to complete more than one-third of the exercise intervention sessions.

### Exercise interventions in patients

#### Supervised (hospital-based) exercise

**Figure 1:**
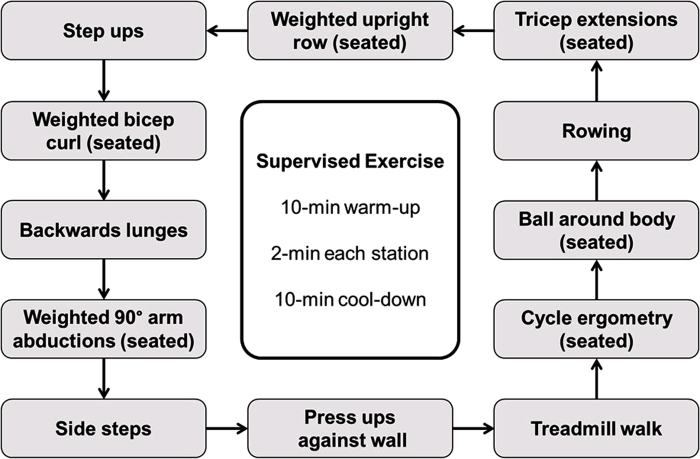
Components of the supervised exercise training therapy

All patients participated in a 10-week senior physiotherapist-led exercise training therapy that consisted of a customised program of aerobic and resistance exercises of the upper and lower limbs (Figure 1). Exercise sessions were held twice weekly (20 sessions in total) using the same calibrated equipment. Each session lasted ~45min and consisted of a 10min seated aerobic warm up, 12 circuit stations each lasting for 2min and a 10min cool down. Patients were encouraged to work into light to moderate pain guided by a four-point subjective scale. If patients reported severe claudication pain, they were advised to change the exercise activity to allow for recovery. Resistance exercises were conducted in the stationary position and were designed specifically to engage upper and lower limb muscle groups. The initial resistance or mass and training device adapted individually to the needs of each patient with careful attention to form (i.e., accommodating full range of motion with the concentric phase synchronised with expiration). The amount of work increased every week, adapted to each case and considering the results obtained by the patient in the previous session, according to the literature.^12^

#### Unsupervised (community-based) exercise

Following successful completion of the supervised exercise therapy, patients were encouraged to continue with a 12-week structured community-based exercise therapy making use of schemes running in local leisure centres. This was designed to be as similar as possible to the supervised therapy in terms of exercise mode, duration, exercise intensity, and frequency and encouraged longer-term improvements in exercise habits; patients were free to adapt this activity according to their preference. Patients received an exercise logbook to record their training sessions and had access to the physiotherapist for feedback and advice if required.

### Measurements

Patient medical history and associated demographics were determined as outlined previously. Remaining measurements were performed at baseline and following completion of the supervised (10 weeks) and unsupervised (12 weeks, 22 weeks cumulative) exercise therapy.

#### Resting heart rate and blood pressure

Patients were rested for 10min in the seated position before measurement of blood pressure via auscultation (Accuson Aneroid Sphygmomanometer, Cirencester, UK). Heart rate (HR) was recorded using an electrocardiogram-calibrated short-range telemetry system (Polar, Kempele, Finland). Mean arterial pressure (MAP) was calculated as one-third [systolic blood pressure (SBP) – diastolic blood pressure (DBP)]+DBP and rate pressure product (RPP) as SBP×HR.

#### Six-minute walking test

Patients performed an overland six-minute walking test (6MWT) according to the standardised protocol outlined by The American Thoracic Society.^15^

#### Walking distance

MWD was defined as the total distance in metres travelled by the patient before claudication pain was so severe they had to stop walking, and the initial claudication distance or PWD as the distance walked before the onset of claudication pain (expressed in both metres and as percentage of the MWD). The rating of perceived exertion (RPE) was recorded using the 6–20 Borg Scale,^16^ and perceived claudication pain (PP) via the claudication 0–4 pain rating scale. $\dot{V}O_{2\mathrm{PEAK}}$ was calculated from the MWD using the generalised prediction equation derived from patients with diverse cardiopulmonary disease standard error of estimate of 1.1ml/kg/min^17^:

$$\dot{V}O_{2\mathrm{PEAK}}\;(\mathrm{ml}/\mathrm{kg}/\min)=4.948+0.023\;\times \;\mathrm{MWD}\;(m)$$

The CRF was estimated for each patient based on the results of the 6MWT and the $\dot{V}O_{2\mathrm{PEAK}}$ calculated using the previous formula.

#### Perioperative risk stratification

In accordance with recent research about assessment of risk of early mortality following abdominal aortic aneurysm repair by preoperative cardiopulmonary exercise testing,^10^ a discriminatory $\dot{V}O_{2\mathrm{PEAK}}$ threshold value of <15ml/kg/min was employed to classify an individual to be at high perioperative risk. The $\dot{V}O_{2\mathrm{PEAK}}$ used for the control group was measured during a cardiopulmonary exercise testing and the $\dot{V}O_{2\mathrm{PEAK}}$ used for the PAD group was calculated from the MWD. In the absence of a dedicated risk model for the PAD patients, the risk model used is based on studies evaluating the morbidity/mortality of patients undergoing elective surgery for abdominal aortic aneurysm.

### Statistical analysis

Data were analysed using the Statistics Package for Social Scientists (IBM SPSS Statistics Version 28.0). Distribution normality was confirmed using repeated Shapiro–Wilk *W* tests. A combination of paired and independent samples *t*-tests were employed to determine the effects of exercise training within and between groups. The number of patients in each group was analysed using chi-square tests to assess how exercise training impacted perioperative risk. Significance for all two-tailed tests was established at *p*<0.05 and data presented as mean±standard deviation (SD) unless otherwise indicated.

## Results

### Compliance

**Figure 2:**
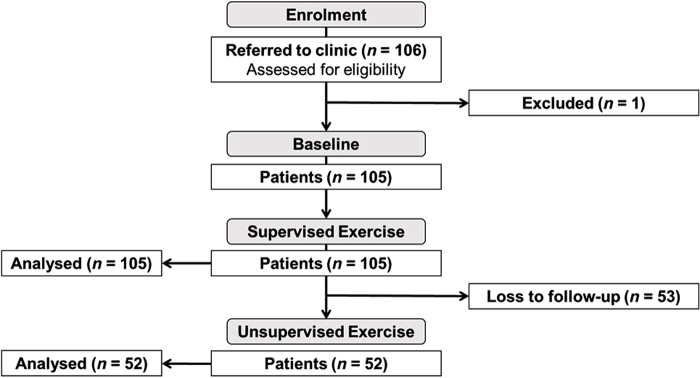
Patient flow. *n*, sample size.

A total of 105 patients (only 1 loss to follow-up) completed the supervised exercise therapy (Figure 2). Of these, 52 patients went on to complete the unsupervised exercise therapy.

### Demographics

Patients who completed the supervised exercise therapy were aged 66±8years old (78% males), compared with the control group, who were 65±8years old with 88% of men. As anticipated, vascular risk factors were more prevalent in the claudication patients (Table 1). Table 2 summarises the severity of their PAD and surgical history.

**Table 1:** Participant and patient characteristics

Group:	Controls	Patients	
Exercise subgroup:	None (*n*=155)	Supervised (*n*=105)	Unsupervised (*n*=52)
Demographics			
Age (years)	65±8	66±8	68±8
Males (*n*/%)	137/88	82/77*	35/67*
Females (*n*/%)	18/12	24/23*	17/33*
Vascular risk factors			
Overweight (*n*/%)	128/83	88/83	43/83
Obese (*n*/%)	79/51	60/57	30/58
Smoker (*n*/%)	48/31	67/63*	30/58*
Diabetes (*n*/%)	10/7	25/24*	11/21*
Hypertension (*n*/%)	8/5	82/77*	41/79*
Dyslipidaemia (*n*/%)	5/3	77/73*	35/67*
Angina/MI (*n*/%)	0/0	42/40*	21/40*
CVA/TIA (*n*/%)	4/3	6/6	3/6
COPD (*n*/%)	0/0	24/23*	11/21*
Medication			
Statins (*n*/%)	5/3	78/74*	38/73*
Aspirin (*n*/%)	4/3	102/96*	50/96*
Warfarin (*n*/%)	0/0	2/2	1/2
Clopidogrel (*n*/%)	0/0	2/2	1/2
Beta-blockers (*n*/%)	8/5	33/31*	12/23*
ACE inhibitors (*n*/%)	0/0	61/58*	29/56*
Calcium channel antagonists (*n*/%)	0/0	49/46*	22/42*
Nitrates/K^+^ATP channel agonists (*n*/%)	0/0	16/15*	8/15*
Diuretics (*n*/%)	0/0	29/27*	18/35*
Antidiabetic medication (*n*/%)	10/7	25/24*	10/19*

Values are mean±SD or frequency (n)/percentage (%). ACE = angiotensin converting enzyme; COPD = chronic obstructive pulmonary disorder; CVA = cerebrovascular accident; MI = myocardial infarction; TIA = transient ischaemic attack.

*p<0.05 vs controls.

**Table 2:** Peripheral artery disease demographics

Subgroup:	Supervised (*n*=105)	Supervised+Unsupervised (*n*=52)
Previous surgery		
Lower extremity revascularisation (*n*/%)	27/25	17/33
Laterality		
Unilateral lesions (*n*/%)	48/45	19/36
Bilateral lesions (*n*/%)	57/54	33/64
Bilateral:Unilateral (AU)	1.18	1.74
Distribution		
Affected limbs (*n*)	163	85
Single segment (*n*)	61	37
Multiple segments (*n*)	102	48
Multiple:Single (AU)	1.67	1.30

Values are frequency (*n*)/percentage (%). AU = arbitrary units.

### Cardiorespiratory fitness

**Figure 3:**
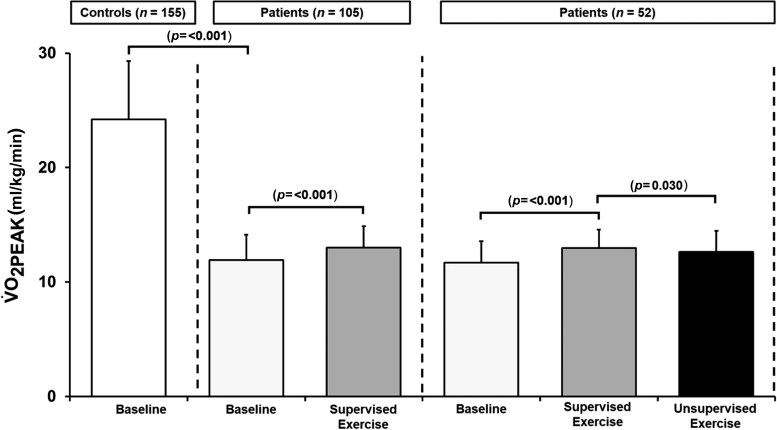
Cardiorespiratory fitness before and in response to supervised and unsupervised exercise training. Values are mean±SD.

At baseline, estimated CRF of patients was 50% lower than that of controls ($\dot{V}O_{2\mathrm{PEAK}}$=12±2ml/kg/min vs 24±5ml/kg/min, *p*=<0.001, Figure 3) highlighting how poorly conditioned they were. $\dot{V}O_{2\mathrm{PEAK}}$ increased following supervised exercise (+1.1±1.4ml/kg/min; *p*=<0.001, Figure 3). In patients who subsequently completed unsupervised exercise, $\dot{V}O_{2\mathrm{PEAK}}$ decreased (−0.3±1.1ml/kg/min, *p*=0.030; Figure 3) though it remained elevated compared with baseline pretraining values (*p*=<0.001). Patient BP, HR, RPE and PP remained unchanged following supervised and unsupervised exercise (all *p*=>0.05, Table 3).

**Table 3:** Cardiorespiratory and ambulatory responses to exercise training

Subgroup:	Supervised (*n*=105)		Supervised+Unsupervised (*n*=52)		
Timepoint:	Pre	Post	Pre	Post^(Supervised)^	Post^(Supervised+Unsupervised)^
Cardiopulmonary (Rest)					
HR (b/min)	70±12	72±11	69±10	71±11	73±12
Δ (b/min)		2±8		2±6	3±8
SBP (mmHg)	147±20	145±19	144±15	147±19	145±18
Δ (mmHg)		−3±22		3±22	1±17
DBP (mmHg)	80±12	78±11	80±10	81±12	79±13
Δ (mmHg)		−3±12		0±13	−1±15
MAP (mmHg)	102±13	100±13	101±9	102±13	101±13
Δ (mmHg)		−3±14		1±14	0±13
RPP (×10^3^ AU)	10.3±2.1	10.4±2.2	10.0±1.5	10.5±2.2	10.5±2.2
Δ (×10^3^ AU)		0.1±0.2		0.5±1.9	0.5±2.1
Ambulatory (Exercise)					
PWD (m)	127±90	170±108*	116±77	149±81	159±75*
Δ (m)		44±81*		33±81	44±88*
PWD (%)	38±23	45±24*	37±25	41±19	47±20
Δ (%)		8±21*		4±24	10±24
MWD (m)	304±95	348±86*	293±82	349±70*	334±80*^,†^
Δ (m)		44±71*		56±58*	41±72*^,†^
RPE (AU)	10±3	11±3	10±3	11±2	12±3
Δ (AU)		0±3		1±3	1±4
PP (AU)	2±1	2±1	2±1	2±1	2±1
Δ (AU)		0±1		0±1	0±1

Values are mean±SD. Δ is change calculated from baseline (Pre). AU = arbitrary units; HR = heart rate; MAP = mean arterial pressure; MWD = maximum walking distance; PP = perceived claudication pain; PWD = pain-free walking distance; RPE = rating of perceived exertion; RPP = rate pressure product; S/DBP = systolic/diastolic blood pressure.

*p<0.05 vs Pre within subgroup.

^†^*p*<0.05 vs Post^(Supervised)^ within subgroup.

### Maximum walking distance

Supervised exercise increased MWD by 24±41% (+44±71m, *p*=<0.001), whereas a decrease was observed following unsupervised exercise (−15±48m, *p*=0.030, Table 3), albeit still elevated above baseline (*p*=<0.001).

### Pain-free walking distance

PWD increased following supervised exercise (+44±81m, *p*=<0.001; Table 3) and was maintained following unsupervised exercise (+11±91m, *p*=0.572; Table 3). Males recorded a more marked increase in PWD, compared with females (+58±78m vs +4±83m, *p*=0.043; Table 4), whereas obese patients recorded a lower increase in PWD (+24±77m vs +74±81m; *p*=0.022; Table 4), compared with non-obese patients.

**Table 4:** Supervised exercise responses according to cardiovascular risk factors

Independent variable		MWD m	$\dot{V}$O_2PEAK_ ml/kg/min	PWD m
Sex	♂	+49 ±64	+1.14 ±1.48	+58 ±78^‡^
	♀	+24 ±89	+0.56 ±2.05	+4 ±83^‡^
Obesity	+	+43 ±80	+0.99 ±1.84	+24 ±77^‡^
	−	+45 ±58	+1.03 ±1.34	+74 ±81^‡^
CVA/TIA	+	+13 ±37	+0.31 ±0.85	−22 ±80
	−	+46 ±72	+1.05 ±1.66	+52 ±80
COPD	+	+20 ±99	+0.45 ±2.27	+37 ±64
	−	+51 ±60	+1.17 ±1.37	+48 ±88
Revascularisation	+	+49 ±69	+1.13 ±1.59	+73 ±79
	−	+42 ±72	+0.96 ±1.66	+25 ±79
Statins	+	+36 ±70*	+0.82 ±1.61^†^	+50 ±85
	−	+67 ±70*	+1.53 ±1.61^†^	+32 ±75
CCA	+	+31 ±74	+0.71 ±1.71	+39 ±110
	−	+55 ±67	+1.26 ±1.54	+49 ±63

Values are mean±SD. Data expressed as the absolute change relative to baseline. CCA = calcium channel antagonists; CVA = cerebrovascular accident; COPD = chronic obstructive pulmonary disorder; MWD = maximum walking distance (*n*=105) ; PWD = pain-free walking distance (*n*=58); TIA = transient ischaemic attack; $\dot{V}$O_2PEAK_ = oxygen uptake (*n*=105).

*p<0.05 vs given subgroup for MWD.

^†^*p*<0.05 vs given subgroup for $\dot{V}$O_2PEAK_.

^‡^*p*<0.05 vs given subgroup for PWD.

### Perioperative risk

**Figure 4:**
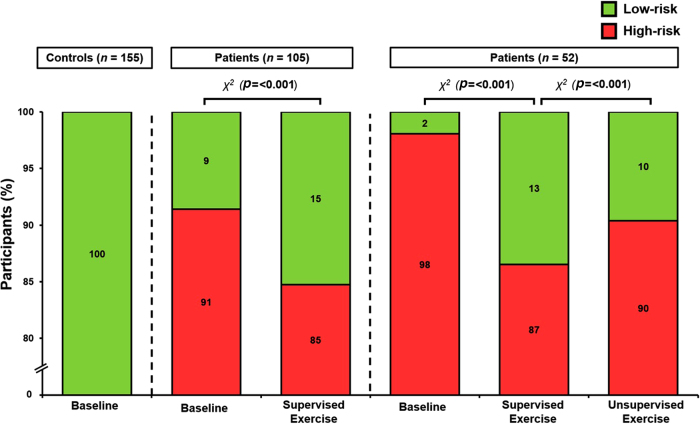
Perioperative risk stratification. Risk stratification based on peak oxygen uptake ($\dot{V}O_{2\mathrm{PEAK}}$) boundary threshold of <15ml/kg/min (high-risk) and >15ml/kg/min (low-risk).^10^ χ, chi-square.

At baseline, 91% of patients were classified as high perioperative risk given a $\dot{V}O_{2\mathrm{PEAK}}$ <15ml/kg/min, whereas all controls were considered low risk (Figure 4). Supervised exercise and the corresponding improvement in estimated CRF reduced perioperative risk (−6%, *p*=<0.001; Figure 4). Unsupervised exercise failed to maintain this improvement and increased perioperative risk (+3% vs supervised exercise, *p*=<0.001; Figure 4), although still below baseline (*p*=<0.001).

## Discussion

The present study has identified three important findings. First, it puts into clear perspective precisely how physically deconditioned PAD patients with IC are relative to age- and sedentary-matched healthy controls. Second, supervised and, to a lesser extent, unsupervised exercise, improves functional walking capacity and CRF, responses that were suppressed in females and as a function of obesity. Third, from a practical perspective, the exercise-induced improvement in CRF translated into a marked reduction in perioperative risk, which has important clinical implications for patient care and management.

A subjective assessment of a patient’s functional capacity by an experienced clinician forms the primary component of preoperative evaluation,^18^ whereby diminished capacity increases the likelihood of complications and mortality following surgery.^19^ In contrast, the more objective, formal assessment of CRF using established cardiopulmonary metrics including VO_2PEAK_ allows for a more accurate measure of whether a patient will tolerate major surgery.^20^ In the present study, PAD patients were defined by extremely low basal CRF that was ∼50% lower than ‘already’ poorly conditioned physically inactive, albeit healthy, controls, highlighting that our patients were even more deconditioned than pre-existing literature suggests.^12^ This difference is possibly related to the older age and increased severity of PAD and associated comorbidities exhibited by the patients in our study compared with those included in previous RCTs.^21^

Importantly, our findings confirm the therapeutic benefits conferred by supervised exercise training for PAD patients with IC as indicated by marked increases in walking capacity and CRF, reflected by functional improvements in MWD, PWD and $\dot{V}O_{2\mathrm{PEAK}}$, consistent with previous research.^4^ While the underlying mechanisms for these improvements are likely multifactorial, an increase in pain tolerance and improvements in redox status comprising increased vascular nitric oxide bioavailability facilitated by an ‘upstream’ reduction in free radical-mediated oxidative stress and inflammation are thought to play pivotal roles.^22-24^ A recent review shows that exercise positively modulates pathways related to inflammation and the atherosclerotic process and may attenuate the progression of lower limb myopathy.^25^ It should also be noted that our data confirm previous research,^25,26^ whereby exercise training was less effective in obese patients and women, further supporting the development of arguably more ‘targeted’, intensive exercise training interventions for these populations. Furthermore, our findings encourage the notion that supervised exercise training should be prioritised over ‘go home and exercise’ advice. The reduction in MWD and CRF, and corresponding increase in perioperative risk (+3%) observed following unsupervised exercise was likely attributable to a lower intensity or frequency of exercise, which is often observed during unsupervised home-based exercise.^27^ If supervised exercise training is not feasible, previous research has suggested that regular meetings or phone calls can be effective for maintaining patient adherence to an exercise protocol to counter this decline in functional capacity.^28,29^

A ‘high-risk’ surgical patient, accounts for ∼13% of cases yet contributes to a disproportionate >80% of all postoperative complications and mortality.^30^ A high-risk patient, defined by a $\dot{V}O_{2\mathrm{PEAK}}$<15ml/kg/min, is at increased risk of death within 30–90 days of elective abdominal aortic aneurysm (AAA) repair.^10^ At baseline, 91% of our PAD patients were considered high risk. Their risk decreased with supervised exercise and was further maintained following the unsupervised exercise intervention. These results encourage the systematic prescription of exercise training for PAD patients with IC before, or indeed as an alternative to, surgical revascularisation. Future studies should look to further stratify/optimise perioperative risk in this specific population using cardiopulmonary exercise testing to directly assess $\dot{V}O_{2\mathrm{PEAK}}$ and associated metrics.^31^

We acknowledge that this study has some limitations, constrained in part by the unavoidable challenges associated with patient recruitment, compliance, and access to specialist infrastructure/resources in the hospital (NHS) setting. First, we were logistically unable to directly perform cardiopulmonary exercise testing for a direct assessment of cardiorespiratory fitness ($\dot{V}O_{2\mathrm{PEAK}}$) in PAD patients, owing to limited resources and select differences in the care pathway for these patients. Instead, we opted to assess $\dot{V}O_{2\mathrm{PEAK}}$ indirectly using a validation approach. Second, the increase in MWD reported in the current study was relatively low in comparison with the improvements documented following treadmill walking exercise training (∼44m vs 277–326m).^32,33^ This may be related to the fact that the MWD was assessed by a 6MWT—a test that is not designed for this purpose and does not allow comparison with the treadmill. Furthermore, the use of a treadmill is known to overestimate performance and the present study included resistance exercise, which has shown to be inferior to treadmill walking exercise for improving MWD.^34,35^ Third, the perioperative risk prediction model used was based on studies evaluating the morbidity/mortality of patients undergoing elective surgery for AAA. Although vascular patients often have a similar profile defined by a multitude of cardiovascular disease risk factors, the perioperative risk may not be fully generalisable to PAD patients.

In conclusion, these findings advocate for routine prescription of supervised aerobic and resistance exercise, and to a lesser extent unsupervised home-based exercise, both as a treatment option to increase claudication distances and for those patients requiring operative intervention for revascularisation in a bid to reduce perioperative risk in poorly conditioned PAD patients with IC.
